# Efficacy of phosphatidic acid ingestion on lean body mass, muscle thickness and strength gains in resistance-trained men

**DOI:** 10.1186/1550-2783-9-47

**Published:** 2012-10-05

**Authors:** Jay R Hoffman, Jeffrey R Stout, David R Williams, Adam J Wells, Maren S Fragala, Gerald T Mangine, Adam M Gonzalez, Nadia S Emerson, William P McCormack, Tyler C Scanlon, Martin Purpura, Ralf Jäger

**Affiliations:** 1 Human Performance Laboratory, University of Central Florida, Orlando, 32186, FL, USA; 2 Increnovo LLC, 2138 E. Lafayette Pl, Milwaukee, 53202, WI, USA

**Keywords:** Nutritional supplement, Muscle architecture, Ergogenic aid, Phospholipid, Resistance training

## Abstract

**Background:**

Phosphatidic acid (PA) has been reported to activate the mammalian target of rapamycin (mTOR) signaling pathway and is thought to enhance the anabolic effects of resistance training. The purpose of this pilot study was to examine if oral phosphatidic acid administration can enhance strength, muscle thickness and lean tissue accruement during an 8-week resistance training program.

**Methods:**

Sixteen resistance-trained men were randomly assigned to a group that either consumed 750 mg of PA (n = 7, 23.1 ± 4.4 y; 176.7 ± 6.7 cm; 86.5 ± 21.2 kg) or a placebo (PL, n = 9, 22.5 ± 2.0 y; 179.8 ± 5.4 cm; 89.4 ± 13.6 kg) group. During each testing session subjects were assessed for strength (one repetition maximum [1-RM] bench press and squat) and body composition. Muscle thickness and pennation angle were also measured in the vastus lateralis of the subject’s dominant leg.

**Results:**

Subjects ingesting PA demonstrated a 12.7% increase in squat strength and a 2.6% increase in LBM, while subjects consuming PL showed a 9.3% improvement in squat strength and a 0.1% change in LBM. Although parametric analysis was unable to demonstrate significant differences, magnitude based inferences indicated that the Δ change in 1-RM squat showed a likely benefit from PA on increasing lower body strength and a very likely benefit for increasing lean body mass (LBM).

**Conclusions:**

Results of this study suggest that a combination of a daily 750 mg PA ingestion, combined with a 4-day per week resistance training program for 8-weeks appears to have a likely benefit on strength improvement, and a very likely benefit on lean tissue accruement in young, resistance trained individuals.

## Introduction

Phospholipids are a major structural component of all biological membrane systems [1,2]. Phosphatidic acid (PA) or 1,2-diacyl-*sn*-glycero-3-phosphate is a phospholipid that makes up a small percentage of the total phospholipid pool [3-5]. It not only is a constituent of all cell membranes, it also acts as an intermediate in the biosynthesis of triacylglycerols and other phospholipids. It is also suggested to act as an intracellular lipid second messenger that regulates signaling proteins, including several kinases and phosphatases [3,6,7]. One of the signaling proteins that PA has been suggested to stimulate is mammalian target of rapamycin (mTOR) [8,9], a serine threonine kinase that integrates metabolic signals from various factors including protein metabolism and cytoskeleton organization that controls cell growth [10]. Both nutritional and mechanical stimuli have been implicated in stimulating this pathway. These different stimuli appear to act at different substrate levels either upstream or downstream from mTOR. Hornberger and colleagues have suggested that the mechanical activation from external loads (as one may see from a resistance exercise session) may be enhanced with the presence of PA [11]. It has been shown that exogenous supplied PA can stimulate the mTOR pathway via its activation of the substrate S6 kinase [4,7]. Interestingly, the binding of PA to S6 kinase may occur independently of mTOR [12], suggesting that PA may augment the signaling response when mTOR is activated by exercise. These data provide an interesting hypothesis that the ingestion of PA, in combination with a resistance training program, may stimulate potentially greater gains in muscle strength and growth than resistance training alone.

The ability to augment muscle strength and size has important implications for various population groups. Specifically, the ability for a dietary supplement to enhance muscle strength and increase lean mass would be of consequence for competitive athletes who are focused on maximizing strength and size gains, and older adults who are battling the effects of aging and sarcopenia. Presently, there does not appear to be any study available that has examined effect of PA supplementation on strength and lean tissue adaptation. Therefore, it is the purpose of this pilot study to examine if PA ingestion can enhance strength, muscle thickness and lean tissue accruement during an 8-week resistance training program more so than training only.

## Methods

### Subjects

Twenty resistance-trained men (at least 1 year of training experience) volunteered to participate in this randomized, double-blind, placebo-controlled, repeated measures study. None of the subjects were competitive strength/power athletes, but all subjects were currently engaged in recreational weight lifting that included using the squat and bench press exercises. Following an explanation of all procedures, risks and benefits, each subject gave his informed written consent prior to participating in this study. The University Institutional Review Board approved the research protocol. Subjects were asked to not use any anabolic dietary supplements or drugs know to increase muscle and/or performance. Screening for dietary supplements or drugs was accomplished by a health questionnaire filled out during subject recruitment.

Subjects were randomly assigned to one of two treatment groups, 750 mg phosphatidic acid (PA; 23.1 ± 4.4 y; 176.7 ± 6.7 cm; 86.5 ± 21.2 kg) or 750 mg rice flour, which served as placebo (PL; 22.5 ± 2.0 y; 179.8 ± 5.4 cm; 89.4 ± 13.6 kg). Four subjects were dropped from the study. One of the subjects was injured during a recreational activity, another subject dropped out due to a family crisis, and the other two subjects were removed due to a lack of compliance. A total of 7 subjects remained in the PA group and 9 subjects in the PL group. The PA supplement (Mediator™) was obtained from Chemi Nutra (White Bear Lake, MN). Both the PA and PL were in capsule form and were similar in appearance. Subjects were provided a weekly capsule allotment and returned the bottle at the end of the week to receive their next week’s supply. Subjects were required to consume five capsules of either the treatment once per day ad libitum. Timing of capsule ingestion was not controlled. Each capsule contained 150 mg of PA or PL. To standardize post-workout protein ingestion, all subjects were provided a 36-g amino acid and collagen protein blend (see Table 1 for content) mixed in a 500 ml commercial sports drink. This drink was consumed within 30 minutes post-exercise.

**Table 1:** Post-workout amino acid and collagen protein blend ingredients

**Amino acid**	**g AA/100 g of product**	**Amino acid**	**g AA/100 g of product**
Alanine	7.6	Leucine	2.8
Arginine	7.8	Lysine	3.1
Aspartic acid	5.1	Methionine	0.6
Cystine	0.0	Phenylalanine	1.9
Glutamic acid	10.5	Proline	12.2
Glycine	18.2	Serine	2.8
Histidine	1.2	Threonine	1.7
Hydroxylysine	0.5	Tryptophan	0.0
Hydroxylproline	10.8	Tyrosine	0.6
Isoleucine	1.4	Valine	2.0

All groups performed the same 4-day per week, split routine resistance training program for 8-weeks (see Table 2). The subjects were required to exercise with 70% of their 1-repetition maximum (1-RM) for all exercises. The load for the assistance exercises was self-determined by the subject, but they were required to use a load that allowed them to perform a 10–12 RM. A 90-s rest period was required between each set, for all exercises. Subjects trained at their local gym off campus without investigator supervision. However, all subjects maintained a daily training log and turned it in at the end of each week. Feedback to subjects on training logs was provided by certified study personnel. This insured appropriate changes to loading during the 8-week program.

**Table 2:** Eight-week resistance training protocol

**Monday/Thursday**		**Tuesday/Friday**	
**Exercise**	**Sets/Reps (RM)**	**Exercise**	**Sets/Reps (RM)**
Bench Press*	1,4 x 10 – 12	Squats*	1,4 x 10 – 12
Incline DB Press	3 x 10 - 12	Lunge/Front squat	3 x 10 - 12
Seated Shoulder Press*	1,4 x 10 – 12	Leg Curl	3 x 10 - 12
Upright rows	3 x 10 - 12	Knee Extension	3 x 10 - 12
Lateral raises	3 x 10 - 12	Calf Raises	3 x 10 - 12
Shrugs	3 x 10 - 12	Lat Pulldown	4 x 10 - 12
Triceps pushdown	3 x 10 - 12	Seated Row	4 x 10 - 12
Triceps extension	3 x 10 - 12	EZ Bar Curl	3 x 10 - 12
Situps	3 x 25	Dumbbell Curls	3 x 10 - 12
		Situps	3 x 25

### Testing protocol

Subjects reported to the Human Performance Laboratory on two separate occasions. The first testing session occurred prior to the onset of supplementation, while the second testing session occurred at the conclusion of the 8-week supplementation program. All testing sessions occurred at the same time of day, and subjects were requested to maintain a similar daily routine on testing dates. Body composition and *ultrasonography* assessments were performed prior to all strength measures.

### Body composition

Body composition was determined using whole body-dual energy x-ray absorptiometry (DEXA) scans (Prodigy™; Lunar Corporation, Madison, WI). Total body estimates of percent fat, fat and non-bone lean tissue was determined using company’s recommended procedures and supplied algorithms. Quality assurance was assessed by daily calibrations and was performed prior to all scans using a calibration block provided by the manufacturer.

### Strength measures

During each testing session, subjects performed a 1-RM strength test for the squat and bench press exercises. The 1 RM tests were conducted as previously described by Hoffman [13]. Each subject performed a warm-up set using a resistance that was approximately 40-60% of his perceived maximum, and then performed 3–4 subsequent attempts to determine the 1-RM. A 3 – 5 minute rest period was provided between each lift. No bouncing was permitted for the bench press exercise, as this would have artificially increased strength values. Bench press testing was performed in the standard supine position: the subject lowered an Olympic weight lifting bar to mid-chest level and then pressed the weight until his elbows were fully extended. The squat exercise required the subject to rest an Olympic weightlifting bar across the trapezius at a self-chosen location. The squat was performed to the parallel position (that was closely monitored by certified staff), which was achieved when the greater trochanter of the femur was lowered to the same level as the knee. The subject then lifted the weight until his knees were extended. Previous studies have demonstrated good test-retest reliabilities (R > 0.97) for these strength measures [14,15].

### Ultrasonography measurements

Skeletal muscle architecture was assessed on the subject’s self-reported dominant leg using B-mode ultrasound imaging (General Electric LOGIQ P5) with a 12-MHz linear probe. A water-soluble gel was applied to the probe. Images were obtained, as previously described [16], by the same technician for all tests using longitudinal probe positioning. Equal contact pressure was maintained during each measure. Vastus lateralis (VS) fascicle thickness and pennation angle were measured at 50% of femur length over the midbelly of the muscle with the subjects lying in a supine position. Pennation angle was determined by the angle between the deep aponeurosis and the fascicles [17]. Muscle thickness was determined as the distance between the subcutaneous adipose tissue and intermuscular interface. All ultrasonography measures were performed prior to the strength tests. The intraclass correlation coefficients ± SEM for muscle thickness and pennation angle were 0.99 ± .03 and 0.95 ± 0.91, respectively.

### Dietary recall

Three-day dietary records were completed during the week prior to the onset of the study. Subjects were instructed to record as accurately as possible everything they consumed during the day, including between meal and late evening snacks. FoodWorks Dietary Analysis software version 13 (The Nutrition Company, Long Valley, NJ) was used to analyze dietary recalls. Subjects were required to maintain their normal diet throughout the study.

#### Statistical analysis

Seven separate two-way mixed factorial Analysis of Variance (time [PRE, POST] × group [PA and PL]) were used to analyze the body mass (BM), body fat, lean body mass, vastus lateralis thickness and pennation angle, 1-RM bench press and squat data. In the event of a significant F- ratio, Tukey post-hoc tests were used for pairwise comparisons. For effect size, the partial eta squared statistic was reported and according to Green et al. [18], 0.01, 0.06, and 0.14 represents small, medium, and large effect sizes, respectively. An alpha level was set at p ≤ 0.05, and all analyses were performed using PASW version 18.0 (SPSS, Inc., Chicago, IL).

Recent investigations in sport science have suggested that the use of null-hypothesis testing may be inadequate for assessing clinical or practical significance [19,20]. An analysis that infers the magnitude of differences in means may provide a more qualitative interpretation of results. To make inferences on true effects of PA on strength and body composition, a published spreadsheet using the unequal variances t-statistic was used [19]. The effect of PA was calculated as the change score by calculating the difference between the post- and pre-supplementation scores for the PA and PL groups. The precision of the magnitude inference was set at 90% confidence limits, using the p-value corresponding to the t-statistic. The published spreadsheet calculated inferences whether the true population effect was substantially beneficial, harmful, or trivial based on the range of the confidence interval relative to the value for the smallest clinical worthwhile effect. An effect was reported to be unclear if the confidence interval overlapped the thresholds for positive and negative substantiveness (>5% chance that the value was both substantially positive and negative). Or, the chance that the value was positive or negative was evaluated by: <1%, almost certainly not; 1-5%, very unlikely; 5-25%, unlikely; 25-75%, possible; 75-95%, likely; 95-99% very likely; and >99% almost certain. Results were interpreted using magnitude-based statistics, using Cohen’s thresholds (<0.1, trivial; 0.1-0.3, small; 0.3-0.5, moderate; >0.5 large) [20].

## Results

No significant differences were seen in caloric intake between PA (3153 ± 778 kcal) and PL (3387 ± 1168 kcal). In addition, no significant differences were seen in carbohydrate (285 ± 74 g vs. 342 ± 94 g), protein (227 ± 68 g vs. 192 ± 59 g) and fat (125 ± 47 g vs. 136 ± 77 g) intakes between PA and PL, respectively. PA and PL were very well tolerated and no adverse events have been reported.

Pre to post changes in strength, muscle architecture and body composition are depicted in Table 3. Significant main effects (Pre vs. Post) were seen in both 1-RM bench press (p = 0.006) and 1-RM squat (p = 0.001) for both groups combined. However, no significant interactions were observed. A significant main effect was also observed for vastus lateralis thickness (p = 0.001), but not for pennation angle (p = 0.156). No significant interactions were noted in either variable. No change in body mass (p = 0.253) was seen following eight weeks of training in either group, but a significant main effect was noted in the change in lean body mass (p = 0.045). A trend (p = 0.065) towards a significant interaction was observed for in lean body mass. The post hoc power analyses (Table 4) ndicated that values ranged from 0.05 to 0.46 for all group X time interactions and 0.05 to 0.97 for main effects for time.

**Table 3:** Strength, muscle architecture and body composition changes

**Variable**	**Group**	**PRE**	**POST**
1-RM Bench Press (kg)	PA	122.1 ± 21.6	128.3 ± 21.6
	PL	115.2 ± 29.6	119.0 ± 28.6
1-RM Squat (kg)	PA	134.5 ± 44.1	151.6 ± 41.1
	PL	138.9 ± 32.9	151.8 ± 33.9
Vastus Lateralis Thickness (cm)	PA	2.10 ± 0.43	2.41 ± 0.27
	PL	1.94 ± 0.41	2.24 ± 0.54
Vastus Lateralis Pennation angle (°)	PA	16.49 ± 2.95	18.34 ± 3.09
	PL	15.6 ± 3.28	16.7 ± 4.21
Body Mass (kg)	PA	86.5 ± 21.2	88.0 ± 18.9
	PL	89.4 ± 13.6	89.5 ± 13.4
Body Fat (kg)	PA	15.8 ± 15.4	15.9 ± 13.6
	PL	17.5 ± 9.4	17.5 ± 9.3
Lean Body Mass (kg)	PA	66.2 ± 4.5	67.9 ± 5.6
	PL	68.4 ± 11.2	68.5 ± 11.2

**Table 4:** Statistical estimates for the dependent variables in this study

**Variable**	**p**	**F**	**Effect size**	**Observed power**
1-RM Bench Press (Kg)				
Group x time interaction	0.43	0.60	0.04	0.11
Group Time Effect	0.006*	0.4	0.43	0.85
1-RM Squat (Kg)				
Group x time interaction	0.19	1.92	0.12	0.25
Group Time Effect	0.00*	93.1	0.87	1.0
Vastus Lateralis Thickness (CM)				
Group x time interaction	0.96	0.002	0.00	0.05
Group Time Effect	0.001*	17.1	0.55	0.97
Vastus Lateralis Pennation angle (^o^)				
Group x time interaction	0.69	0.16	0.01	0.07
Group Time Effect	0.16	2.25	0.14	0.29
Body Mass (Kg)				
Group x time interaction	0.35	0.94	0.06	0.15
Group Time Effect	0.53	1.42	0.09	0.15
Body Fat (Kg)				
Group x time interaction	0.99	0.000	0.0	0.05
Group Time Effect	0.95	0.005	0.0	0.05
Lean Body Mass (Kg)				
Group x time interaction	0.065	4.01	0.223	0.46
Group Time Effect	0.045*	4.83	0.256	0.53

**Figure 1:**
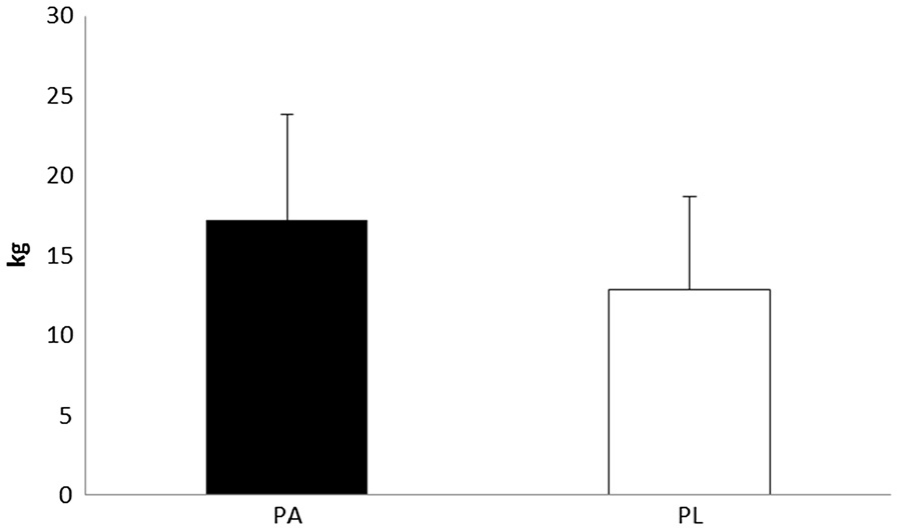
**Changes in Δ 1-RM squat strength.** All data are reported as mean ± SD.

**Figure 2:**
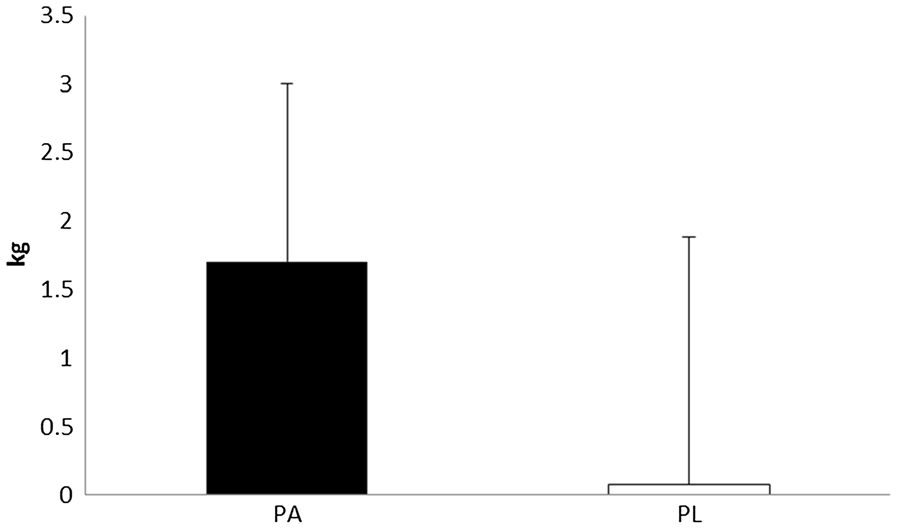
**Changes in Δ lean body mass.** All data are reported as mean ± SD.

Magnitude based inferences on changes in performance and anthropometric measures are described in Table 5. The Δ change in 1-RM squat show a likely benefit from PA on increasing lower body strength. Subjects ingesting PA demonstrated a 12.7% in squat strength, while subjects consuming PL showed a 9.3% improvement (See Figure 1). Improvements in 1-RM bench press were 5.1% and 3.3% in PA and PL, respectively. Magnitude based inferences were unclear regarding any benefit in upper body strength improvements in these subjects consuming the PA. Differences in the changes in muscle architecture (e.g. vastus lateralis thickness and pennation angle) between PA and PL were also unclear. However, it appeared very likely that PA was more beneficial for increasing lean body mass (2.6% increase from pre to post) than PL (a 0.1% change from pre to post) (see Figure 2). Differences in the change in body mass or fat mass between PA and PL were unclear.

**Table 5:** Magnitude based inferences on strength, muscle architecture and body composition changes between groups

**PA vs. PL**	**Mean difference**	**Clinical inference**	**% beneficial/ positive**	**% negligible/ trivial**	**% harmful/ negative**
1-RM Bench Press (kg)	2.38	Unclear	63.5	0	36.5
1-RM Squat (kg)	4.31	Likely	88	4.8	7.2
Vastus Lateralis Thickness (cm)	.007	Unclear	0.25	99.5	0.25
Vastus Lateralis Pennation angle (°)	.79	Unclear	26	18.2	55.8
Body Mass (kg)	.006	Unclear	72	18	10.1
Body Fat (kg)	−14.5	Unclear	50.5	0	49.5
Lean Body Mass (kg)	1.6	Very Likely	96.4	0.7	2.9

## Discussion

This is the first study known that has examined the efficacy of phosphatidic acid on enhancing strength and muscle growth. The results of this study indicate that 8 weeks of supplementation with PA is likely to very likely beneficial in increasing lower body strength and lean body mass, respectively, compared to PL (Table 4). The effects of PA supplementation on upper body strength and muscle architecture were unclear. Recent evidence on rodent models have indicated that resistance exercise or an intermittent muscle stretch can activate mTORC1 by direct binding of PA to mTOR [11,21]. It has been suggested that the mechanical action of muscle contraction can stimulate the growth promoting pathways within muscle [22]. Considering that the mTOR signaling pathway was not examined in this study, we can only speculate on the mechanisms that may have contributed to the observed results.

The mechanical stimulus of resistance training has been demonstrated to be a potent stimulus for increasing protein synthesis [23,24]. If protein or essential amino acids are ingested either before or following a workout, the effect on muscle protein synthesis appears to be magnified [25]. Recent evidence has suggested that leucine, even in low dosages, may be very effective in stimulating muscle protein synthesis [26]. In consideration of the potential effects that protein ingestion has on muscle recovery and remodeling, we felt it important to provide a standardized protein supplement to all subjects (both PA and PL) following each training session. With daily nutritional intake, including protein, similar between each group, the changes noted in this study (increases in lower body strength and lean body mass) likely reflect the ingestion of PA (Tables 3, 4 and 5). Since PA is thought to act on a parallel pathway to a protein stimulus (specifically leucine) on activating the mTOR pathway [8,27], the greater benefit towards increases in lower body strength and lean body mass in PA suggests that the ingestion of this supplement may enhance lean tissue accruement and lower body strength to a greater extent than protein supplementation or resistance exercise only.

Differences between upper and lower body strength gains seen in this study may reflect the training experience of the subjects. Though all subjects had at least one year of resistance training experience, previous research on competitive strength power athletes has indicated that improvements in lower body strength may precede changes in upper body strength [28,29]. This may reflect a greater experience in upper body training and a requirement for performing the squat exercise to appropriate depth and technique. None of the subjects in the study were working with a strength coach or personal trainer prior to their enrollment into the study. Evaluation of the training logs and performance testing were conducted by certified strength and conditioning specialists that reinforced proper technique and form during the testing. Considering the skill and technique necessary for performing the squat exercise, many competitive and recreational resistance trained athletes do not perform this exercise correctly [30]. It is likely that the resistance training experience of the subjects resulted in a relative high level of performance in the bench press exercise. Although all subjects had performed the squat exercise prior to this study, their technical ability and skill for this exercise (i.e. bar placement, knee and foot alignment and lowering to parallel) varied widely. Since proper technique was stressed during the training and testing program it is possible that the subjects had a larger window of opportunity for strength gains based upon improved technique in the squat exercise compared to the bench press exercise. Thus, the strength improvements seen in the squat exercise could be partially attributed to a learning effect.

There were no clear benefits from PA ingestion in changes to muscle architecture of the vastus lateralis (Tables 3 and 5). The training program appeared to result in similar changes in muscle thickness for both groups, but did not result in any significant changes in pennation angle. The results observed in vastus lateralis thickness are similar to those reported by Blazevich and colleagues [31] following 5-weeks of training in competitive athletes, but greater than those reported by Santilla and colleagues [32] following 8-weeks of training in tactical athletes. However, the subjects in the latter study were also performing their basic military training that likely blunted maximal muscle growth. Comparisons between studies are also difficult to make due to the differences in subjects training status, the resistance training program and training duration. Although PA did appear to have a likely benefit on 1-RM squat changes, it did not have a similar effect on changes in vastus lateralis thickness. A recent study has questioned the importance of vastus lateralis changes on lower body strength performance as those investigators were unable to find any significant correlation between vastus lateralis thickness and lower body power performance [33]. The lack of any significant changes in pennation angle for either group may also be related to resistance training experience, as experience does appear to impact the magnitude of change in pennation angle [31].

There are a number of limitations associated with this study. The scientific treatise that has emanated on phosphatidic acid and its role on muscle protein synthesis stimulated the desire to examine this further. Although the results of this study provide a degree of efficacy on this novel ingredient, it does not provide any support to the previously discussed mechanisms of action. However, the results of this study do provide some evidence on the proof of concept that PA may have a role in muscle strength and lean tissue accruement. Additional research is needed to add support to these results: a bioavailability study to investigate the absorption profile of orally administered PA, a muscle biopsy study to investigate the potential increase in muscle PA content, different target groups: trained, untrained, elderly subjects, dose finding studies to investigate if the effect of PA is dose dependent, the minimum effective dose and mechanistic studies. This will have important implications for athletes participating in strength/power sports, as well as mature adults attempting to maintain muscle strength and mass as they age.

In conclusion, the results of this study suggest that a combination of a daily 750 mg PA ingestion, combined with a 4-day per week resistance training program for 8-weeks appears to have a likely benefit on strength improvement, and a very likely benefit on lean tissue accruement in young, resistance trained individuals. Additional research is warranted to provide further elucidation on the mechanisms that govern PA and muscle protein synthesis, muscle growth and performance.

## Competing interests

MP and RJ have been named as inventors on pending patents by Chemi Nutra. MP and RJ are independent paid consultants to Chemi Nutra. All other authors declare that they have no competing interests.

## Authors’ contributions

JRH was the primary investigator, supervised all study recruitment and data/specimen analysis. JRH, MP and RJ designed study, JRH and JRS performed the statistical analysis, JRH supervised the manuscript preparation, JRS, DRW and RJ helped drafting the manuscript. DRW, AJW, MSF, GTM, AMG, NSE, WPM and TCS assisted with data collection and data analysis. All authors read and approved the final manuscript.
